# Health Behavior Knowledge and Self-efficacy as Predictors of Body Weight

**DOI:** 10.4172/2161-0509.1000169

**Published:** 2015-09-15

**Authors:** Pouran Faghri, Jennifer Buden

**Affiliations:** Department of Allied Health Sciences, University of Connecticut, Storrs, CT, USA

**Keywords:** Self-efficacy, Obesity, Stress management

## Abstract

Obesity is a public health concern with significant economic costs affecting employers. Worksite wellness programs benefit from developing tailored interventions that consider employees’ health-related knowledge and self-efficacy to change behavior. Correction is a high stress occupation with elevated rates of overweight and obesity. Poor stress management and barriers to achieve optimal health in the work environment increases the need for adequate knowledge and self-efficacy, or the level of confidence to eat healthy and be physically active. This cross-sectional pilot study used a sample of sixteen correctional employees who participated in a Nutrition and Physical Activity Questionnaire. This survey assesses knowledge and self-efficacy for nutrition and physical activity and current health behaviors, such as current dietary habits and level of physical activity. Demographic and anthropometric data were also collected for statistical analyses. Participants were primarily male correction officers working first shift with a mean (±SE) BMI of 29 (±1.05) kg/m^2^, classified as overweight. Multiple regression analyses revealed that knowledge and self-efficacy scores predicted variation in BMI when controlling for other scores in the model. Findings from this study may be applicable for future health promotion interventions in high-risk occupations. In high-risk occupations such as corrections, understanding environmental and organizational barriers to achieving good health and reducing chronic disease risk is important. However, in addition to reducing these barriers, increasing knowledge, improving skills and self-efficacy to achieve good health are also critical in order to develop effective interventions for this population.

## Introduction

Obesity is a national epidemic in the United States that affects all levels of the population. Sixty-nine percent of adults over the age of 20 are overweight or obese according to data collected in 2011-2012 by the Centers for Disease Control and Prevention. Further, obesity rates continue to be a concern in children and adolescents [1]. Nutrition and exercise habits remain key components to promote healthy behaviors among all age groups.

Overweight and obesity have a multitude of consequences beyond increased risk for comorbidities, including economic, social, and societal costs. For workplaces with elevated rates of overweight and obesity, the associated higher rates of presenters, absenteeism and low productivity, have a significant economic impact on employers [2]. Several studies have identified positive relationships between obesity, absenteeism [3,4] and low productivity [5]. Furthermore, there is a positive association between job characteristics in a high stress, low control workplace, such as exposure to unpredictable situations during work hours or having a lack of control over job routines, and BMI [6-9]. Corrections officers have higher rates of overweight (40.7%) and obesity (43.3%) compared to national norms in the United States where 33.6% of adults are overweight and 34.9% are obese, respectively [10-13]. Evidence has shown that employees may cope with job stress by developing unhealthy behaviors such as decreased physical activity and unhealthy eating, which may contribute to cardiovascular disease and type II diabetes [14].

As employers are required to compensate for portions of rising healthcare costs, workplace wellness programs have become a feasible approach to improve health and wellness in the workplace. Benefits of these programs extend to both employees and employers. A meta-analysis on the return on investment from prevention and wellness programs in the workplace revealed that employers see significant savings from both medical costs and reduced absenteeism [15]. Beyond the immediate health benefits for employees, research shows that wellness programs also improve organizational morale and job satisfaction [15,16]. With the reported higher health care costs linked to overweight and obesity, workplace wellness programs have gained attraction in recent years as a cost savings approach. However, most employers institute a “one size fits all” approach rather than a tailored intervention that considers overweight and obese employees’ knowledge, attitude and confidence to manage their body weight in a high stress work environment [17].

Numerous factors contribute to the obesity epidemic, including environment, genetics, socio-demographics, and cultural influences, such as knowledge and attitude [18]. Characteristics of the job may increase the need for adequate knowledge of healthy lifestyle behaviors that contribute to weight gain. For example, lack of access to healthy foods in the workplace and unique job stress may make it more challenging to engage in healthy behaviors if coping mechanisms and knowledge are inadequate. Understanding the relationships between knowledge, attitude and health behaviors related to dietary intake, physical activity and body weight could play an important role in developing intervention strategies targeting energy balance and reducing the prevalence of obesity.

The purpose of this pilot study was to investigate the relationships between obesity-related behaviors (diet, physical activity) and health behavior knowledge and attitudes (self-efficacy for eating and exercise) in employees working in a high stress occupation. We hypothesize that there is a negative relationship between nutrition and physical activity knowledge, self-efficacy and BMI. Those with less knowledge and self-efficacy scores will have higher BMI.

This information may help identify knowledge and attitude barriers to physical activity and healthy eating at the workplace and aid in developing tailored educational materials and interventions for overweight and obese employees.

## Methods

### Study design

This was a cross-sectional study of a survey called the Nutrition and Physical Activity Questionnaire (NPAQ) [19] completed by a group of correctional employees.

#### Measurements

##### Nutrition and physical activity questionnaire

The NPAQ developed by Faghri et al. [19] consists of 67 multiple-choice items divided into four sections and takes approximately 35-40 minutes to complete. Higher sum scores within each domain are indicative of greater knowledge, self-efficacy and healthier behaviors. Section I, Nutrition Knowledge, contains 49 multiple-choices items to assess understanding of cholesterol, fat, fiber, sodium and protein in the diet as well as knowledge of dietary guidelines, Nutrition Facts label reading, and weight management. Items are true/false, application, or best choice questions. Each question is valued at one point for a correct answer and zero points for incorrect answers. The total possible score is 97. Questions were adapted from Hawkes et al., Parmenter et al., Fielder, and Mackison et al. [20-23]. Section II, Eating Assessment, contains 8 multiple-choice items to assess average intake of fruits, vegetables, whole grains and low-fat dairy products. Respondents can obtain up to 3 points per item, where a score of 0 points for an item indicates being not at all healthy and a score of 3 points indicates being mostly healthy/extremely healthy. Questions were adapted from Fielder [22] and the total possible score is 24.

Section III, Physical Activity Knowledge and Physical Activity Assessment, contains 6 multiple-choice items to assess understanding of activity guidelines to obtain health benefits. Each question is valued at one point for a correct answer, with a total possible score of 6. Two additional items assess self-reported frequency and type of physical activity over the past week (mild, moderate and vigorous) for at least 30 minutes per day. Questions were adapted from Steptoe et al., Morrow et al., and Washburn et al. [24-26]. Section IV, Self-efficacy, contains 8 items to measure exercise self-efficacy (ESE) and 12 items to measure weight loss self-efficacy (WLSE). Questions for this domain follow a Likert scale ranging from “Not Confident” (1 point) to “Very Confident” to “I already do this” (5 points). The total possible score for ESE is 40, and for WLSE is 60. Questions were adapted from Sallis et al. [27]. Sum scores from each domain were used to create new observed variables for statistical analyses.

##### Demographic information

Age, gender, race/ethnicity, level of education, job title at DOC, shift, years worked at DOC, income, reported hours of voluntary overtime worked and reported hours of mandatory overtime worked were also collected. A Registered Dietitian Nutritionist and Research Assistants assessed height and weight during survey distribution to calculate BMI (in kg/m^2^) and waist circumference (in cm).

### Study population

Sixteen correctional employees from one correctional institution completed the NPAQ.

### Statistical analyses

Data was analyzed using IBM SPSS^™^ software version 21 for descriptive analysis and SAS version 9.3 for advanced statistical analyses. The primary variables analyzed include BMI (kg/m^2^), waist circumference (cm), age (years), job classification, gender, and assessment scores from the NPAQ. New variables were computed using sum scores from each domain in the NPAQ. Descriptive statistics were run on demographic variables and sum scores to obtain measures of central tendency (mean, standard deviation, median) and frequency distributions. Normality was assessed using histograms and numerical tests to evaluate p values. Prior to running individual inference tests, the appropriate test assumptions were run. ANOVAs and regressions were run to evaluate statistical significance among sum scores for dependent variables (gender, BMI, job position). Further, post hoc testing was performed to identify where significance lies, when necessary. Cut-offs for significance were set at p < 0.05.

## Results

### Descriptive statistics

The majority of participants were overweight, with a mean [standard error (SE)] BMI of 29.03 kg/m^2^ (1.05) and waist circumference of 97 cm (3.93), measured at the time the survey was distributed. Participants were primarily male (68.75%), and had a mean age of 42. Mean scores for nutrition knowledge and physical activity knowledge were 56.125 and 4.687, respectively. Mean scores for weight loss self-efficacy and exercise self-efficacy were 42.437 and 28.812, respectively. The majority of participants classified themselves as being White, European descent (50%) and had an average education attainment of some college (40%). Distribution of respondents’ job class included, 75% Correction Officers (CO’s), 12.5% Lieutenants, and 12.5% Support Staff (maintenance, food service, admin staff, teacher, chaplain). The majority of participants worked at the Department of Corrections (DOC) for 6-10 years (50%), and worked on first shift (62.5%). (Table 1) provides further anthropometric and demographic information.

A moderate negative skew for physical activity knowledge score was noted. All other scores assessing knowledge and self-efficacy were normally distributed (nutrition knowledge, eating assessment score, exercise self-efficacy, and weight loss self-efficacy).

Simple linear regressions to evaluate knowledge and self-efficacy scores as predictors of BMI revealed no significance, indicating that these scores independently do not predict BMI (Table 2). However, further analysis using multiple linear regression revealed that 58% of the variance in BMI could be accounted for by knowledge (nutrition, physical activity) and self-efficacy scores (ESE, WLSE) (p=0.035). The adjusted R-squared for this model indicated that 43% of the variance in BMI was predicted by these variables. Evaluation of the coefficients indicated that for every unit increase in physical activity knowledge score, BMI would decrease by 3.58, when controlling for the other variables in the model (p=0.05). For every unit increase in WLSE, there would be an expected 0.39 decrease in BMI, when controlling for the other variables (p=0.008). To provide better interpretation of the strength of these outcomes, standardized beta coefficients were run for the variables within the model. Weight loss self-efficacy had the highest standardized coefficient (−0.694) and one could infer that a one standard deviation decrease in WLSE would yield a 0.694 standard deviation predicted increase in BMI (Table 3).

## Discussion

This pilot study provides strong findings related to the associations between BMI, health behaviors and the influence of knowledge and self-efficacy surrounding obesity in a high stress occupation such as corrections. Unhealthy lifestyle behaviors contribute to obesity risk. Having knowledge and skills to engage in healthy behaviors raises confidence to overcome barriers and increases perceived benefits to change behavior. Despite the growth of worksite health promotion, corrections remains an occupation of concern due to high obesity rates, high levels of stress, and lack of job control that indirectly influence lifestyle behaviors [28,29]. Social Cognitive Theory (SCT) proposes there is a relationship between knowledge, attitude, and skills, which translates into action or behavior by raising confidence to overcome barriers. Previous health promotion research has used SCT as a model for evaluating the relationship between knowledge, self-efficacy, and health behavior [30].

A previous study in corrections reported that employees with higher levels of obesity (BMI) had lower self-efficacy to engage in healthy behaviors [10]. Associations between social-cognitive variables, health behaviors and outcomes have also been reported [31,32]. However, no previous research has evaluated knowledge or self-efficacy as predictors of current health behavior (dietary habits, level of physical activity) or weight status. In addition, little research has evaluated the interaction effect between knowledge and self-efficacy on weight status. The results from this cross-sectional analysis provided significant findings to better understand the corrections population, a group at high-risk for obesity and its’ associated comorbidities. From multiple regression analyses, we can conclude that some cognitive scores are associated with BMI in corrections employees. Our model indicated that both physical activity knowledge and weight loss self-efficacy are associated with BMI, when controlling for other factors. However, violations of some regression assumptions indicate we must be cautious making conclusions about these results. In addition, because this was only a cross-sectional analysis, we can only infer that these cognitive variables are associated, and we are unable to predict a causal relationship. Future research should use a larger sample size to test statistical assumptions and evaluate generalizability of results.

A quasi-experimental weight loss intervention using a participatory design used a modified version of the NPAQ. They administered the survey at baseline and 20 weeks to measure change in knowledge and self-efficacy for nutrition, physical activity, and dietary habits. When controlling for gender and age, some change in body weight and waist circumference were explained by nutrition knowledge and exercise self-efficacy. This study suggests that improving knowledge and self-efficacy would influence the effectiveness of an intervention in the worksite setting [11]. Similar studies have evaluated knowledge and self-efficacy to predict participation in a worksite health promotion program, and suggest that individuals with lower knowledge or self-efficacy are less likely to participate or adhere to interventions [33-35].

Despite that some significant results were identified; this study is limited by its small sample size. Future implications of this research include: 1) evaluation of these measures with a larger sample size to better understand this population and 2) development of tailored interventions incorporating nutrition and physical activity knowledge and health behavior attitude and self-efficacy to aid sustainable behavior change.

## Conclusion

Chronic diseases remain a national public health concern, and the worksite environment is an appropriate setting to provide tailored interventions by targeting multiple levels that influence lifestyle behaviors. Dietary habits and level of activity mediate chronic disease risk by aiding in weight management. Correctional employees may face additional barriers to engage in healthy lifestyle behaviors that mitigate chronic disease risk. Rotating shifts, understaffing, high levels of stress, low job control, work-family conflict, and other work culture factors may reduce an individuals’ motivation to engage in healthy behaviors. This research is significant because it assesses health behavior constructs that contribute to level of obesity in a high-risk population. Social-cognitive variables such as knowledge and self-efficacy are critical in planning interventions to develop effective health promotion strategies that have the greatest impact. Future research should evaluate these variables with a larger sample size and perform advanced statistical analyses using structural equation modeling to look at cognitive scores and behavior as predictors of body weight.

## Figures and Tables

**Table 1:** Anthropometrics and demographics of study participants.

Gender	Male	68.75% (n=11)
	Female	31.25% (n=5)
Age	Years ± SD	42.06 ± 8.94
Anthropometrics	Weight: pounds ± SE	194.64 ± 10.29
	BMI: kg/m^2^ ± SE	29.02 ± 1.05
	BMI (males): kg/m^2^ ± SE	30.24 ± 1.02
	BMI (females): kg/m^2^ ± SE	26.34 ± 2.20
	Waist Circumference (males): cm ± SE	104.9 ± 2.14
	Waist Circumference (females): cm ± SE	81.4 ± 2.83
Race	White, European Descent	50%
	Black, African American, African	25%
	American Indian, Alaska Native	0%
	Asian, Asian American (includes Filipino, Korean, Chinese)	0%
	Other	18.75%
	Latino or of Hispanic Origin or Descent (answered yes includes: Puerto Rican, Cuban American, Mexican American, etc.)	18.75%
Education	Less than high school	6.67%
	High School Graduate or GED	26.67%
	Some College	40.00%
	College Degree (2 or 4 year)	26.67%
	Graduate Degree	0%
Job Class	CO, CTO, Counselor (frontline staff)	75%
	Lieutenant, Captain, Deputy Warden, Warden, Supervisors	12.5%
	Support Staff, Medical Staff	12.5%
Years at DOC	0-5 years	12.5%
	6-10 years	50%
	11-15 years	12.5%
	16-20 years	12.5%
	20 or more years	12.5%
Shift	First	62.5%
	Second	31.25%
	Third	6.25%

CO = Correction Officer; CTO = Correctional Treatment Officer

**Table 2:** Knowledge and self-efficacy scores did not independently predict BMI using simple linear regression. However, relationships revealed by regression coefficients provide relevant interpretations.

*Dependent Variable* = *BMI*				
Independent Variable	R-squared	Regression Coefficient	p value	Interpretation (i.e., could be inferred if there was a significant p value)
Nutrition Knowledge	0.1389	−0.17495	0.1551	For a one unit increase in nutrition knowledge, we would see approximately a 0.2 decrease in BMI
Physical Activity Knowledge	0.0943	−1.62914	0.2473	For a one unit increase in PA knowledge, we would see approximately a 1.6 decrease in BMI
Eating Assessment	0.0043	−0.06361	0.8089	For a one unit increase in eating assessment score (indicating healthy eating), we would see a 0.06 decrease in BMI
Exercise Self-efficacy (ESE)	0.0039	0.06701	0.8176	For a one unit increase in ESE, we would see a 0.06 increase in BMI
Weight Loss Self-efficacy (WLSE)	0.1457	−0.21645	0.1446	For a one unit increase in WLSE, we would see a 0.2 decrease in BMI

**Table 3:** Knowledge and self-efficacy scores predict BMI when controlling for other factors using multiple linear regression (several models tested).

*Dependent Variable* = *BMI*				Parameter Estimates		
Model #	Independent Variables (i.e., predictors)	R-squared	Model p value	Regression Coefficients	p values	Standardized β Coefficient
1	**Nutrition & Physical Activity Knowledge**	0.1919	0.2503			
2	**Nutrition Knowledge & Eating Assessment**	0.1403	0.3743			
3	**Eating Assessment & WLSE**	0.1497	0.3485			
4	**ESE & WLSE**	0.1564	0.3310			
5	**Physical Activity Knowledge & ESE**	0.1291	0.4071			
6	**Nutrition Knowledge**	0.5806	**0.0352**	−0.1564	0.1608	−0.3332
	**Physical Activity Knowledge**			−3.5838	**0.0543**	−0.6755
	**ESE**			−0.2727	0.4012	−0.2551
	**WLSE**			−0.3936	**0.0088**	−0.6941
